# Product Development Partnerships: Delivering Innovation for the Elimination of African Trypanosomiasis?

**DOI:** 10.3390/tropicalmed5010011

**Published:** 2020-01-15

**Authors:** Emma Michelle Taylor, James Smith

**Affiliations:** 1 Department of Social Anthropology, University of Edinburgh, Edinburgh, EH8 9LD, UK; 2 Centre of African Studies, University of Edinburgh, Edinburgh, EH8 9LD, UK

**Keywords:** product development partnerships, research and development, medical innovation, donor policy, African trypanosomiasis

## Abstract

African trypanosomiasis has been labelled as a ‘tool-deficient’ disease. This article reflects on the role that Product Development Partnerships (PDPs) have played in delivering new tools and innovations for the control and elimination of the African trypanosomiases. We analysed three product development partnerships—DNDi, FIND and GALVmed—that focus on delivering new drugs, diagnostic tests, and animal health innovations, respectively. We interviewed key informants within each of the organisations to understand how they delivered new innovations. While it is too early (and beyond the scope of this article) to assess the role of these three organisations in accelerating the elimination of the African trypanosomiases, all three organisations have been responsible for delivering new innovations for diagnosis and treatment through brokering and incentivising innovation and private sector involvement. It is doubtful that these innovations would have been delivered without them. To varying degrees, all three organisations are evolving towards a greater brokering role, away from only product development, prompted by donors. On balance, PDPs have an important role to play in delivering health innovations, and donors need to reflect on how best to incentivise them to focus and continue to deliver new products.

## 1. Introduction

*“Markets work well for society when there is price competition, comprehensive and accurate information, an adequate supply of drugs, where consumers are able to make informed unpressured choices between competing products, and when there are few barriers for entry to market. However, substantial evidence shows that markets have failed to work”*.

[1] (p. 1590)

For the better part of half a century, innovation for the African Trypanosomiases stalled. Following the introduction of drug melarsoprol in 1949, it took 41 years for eflornithine to be registered for the treatment of stage 2 *T. b. gambiense*, and a further 19 years for that drug to be put to full use in a combination therapy (Nifurtimox-Eflornithine Combination Therapy). The Card Agglutination Test for Trypanosomiasis (CATT), launched in 1978, remained the sole screening test for *T. b. gambiense* for over thirty years. And yet, none of these products constituted ‘perfect’ tools, fit for purpose (melarsoprol is dangerous and subject to treatment failure, Nifurtimox-Eflornithine Combination Therapy is expensive and logistically challenging to deliver, and the CATT test lacks sensitivity and cannot provide a confirmatory diagnosis). Rather, a failure of the market and public policy in the years following African independence meant that there was little incentive for the private sector to invest in product innovation for a disease that only affected poor and marginalised communities living along the tsetse belt of Sub-Saharan Africa.

African Trypanosomiasis is often depicted as the quintessential colonial disease [2]. Caused by unicellular protozoan parasites from the genus Trypanosoma, the disease commonly affects humans (*T. b. gambiense* and *T. b. rhodesiense*) and valuable domestic livestock (*T. vivax*, *T. congolense* and *T. brucei* subspecies) [3]. A major epidemic at the start of the twentieth century threatened to obstruct imperial pursuits on the African continent, making trypanosomiases a pan-African priority to unite colonial interests. Neill has documented how the imperative to control Human African Trypanosomiasis (HAT) during this period gave rise to a new medical specialty—tropical medicine—and stimulated the creation of international networks of experts to propose solutions [4]. This networked approach proved successful. By the 1960s, HAT was almost eliminated using a toolkit of interventions devised during the colonial period [5].

Following African independence, basic research into the trypansomiases continued, attracting vast investment from development agencies and their colonial pre-cursors. However, it was the failure to translate this research into new tools and approaches that disappointed [5,6].

Around the turn of the millennium, a HAT epidemic, coupled with a threat to the manufacture and supply of all HAT drugs [7], provided a case example of what Médecins Sans Frontières (MSF) later termed the “fatal imbalance” of research and development for HAT and other diseases of poverty [8]. According to MSF, HAT was not just a neglected disease but, by virtue the market and public policy failings that had rendered it “tool deficient” [8] (p. 13), it was a “most neglected disease” [8] (p. 11). A number of surveys examining the state of the product development pipelines for Neglected Tropical Diseases (NTDs) confirmed this distinction [6,9]. In response, MSF launched the Access to Essential Medicines campaign. Access to Essential Medicines identified three gaps in the drug development process for neglected disease, emphasising the first gap—between basic research and pre-clinical research—as most significant [8]. Access to Essential Medicines argued that in the current profit-driven system, “It is a matter of simple economics: potential return on investment, not global health needs, determines how companies decide to allocate R&D funds” [8] (p. 18). The campaign set out that traditional profit-driven models of innovation could no longer be relied upon to deliver for the world’s poorest and most vulnerable populations. Instead, new models of partnership, driven by patient needs, were urgently required. This position has since gained widespread support in light of the augmented demands posed by disease elimination goals [10].

International development, and global health in particular, has recognised the role of partnerships to deliver impact. The Millennium Development Goals recognised the role of partnerships in ensuring development activity was harmonised, locally relevant and mutually accountable. The current Sustainable Development Goals emphasis partnerships even more strongly [11]. But partnerships can and do have many meanings. There is a broad literature exploring the realities of partnerships in international development and global health, mainly around the extent to which they are truly reciprocal or equal, but this is beyond the scope of this paper [12,13]. For our purposes, we define a partnership as ‘a collaborative relationship among organisations in which risks and benefits are shared in pursuit of a common goal that would not otherwise be attainable’.

A partnership model prioritising a need-based approach to research and development (R&D) is now operational, delivering for a host of neglected tropical and zoonotic infections previously underserved by the market. It is known as a Product Development Partnership (PDP). While different permutations exist, certain key features define PDPs: they are “not-for-profit organisations that drive product development for neglected diseases in conjunction with external partners;” they “are formal organisations rather than one-off collaborations between public and private groups and share a number of characteristics in the way they work. They focus on one or more neglected diseases and develop products suitable for use in developing countries” and “they generally use private sector management practices” in their R&D activities [14] (p. 116). For the African trypanosomiases, PDPs are proving to be a game changer, with some examples delivering not just single products but product pipelines for the first time in history.

Today, *T. b. gambiense* stands on the cusp of elimination (in 2017 just 1447 new cases were reported [15]). Yet, product development for this disease continues unabated. PDPs have already contributed to the global campaign to see HAT eliminated as a public health problem by 2020 —delivering new diagnostics and treatments—but their larger contribution is yet to be realised: delivering at scale the products that could see the transmission of HAT eliminated by 2030. Reaching and sustaining this goal requires HAT services, which have traditionally been run vertically, to be integrated into national healthcare structures. This paradigm shift is only made possible by the development of new, safe, easy to use products that can deployed at point of need (in addition to the active participation of public bodies in disease endemic countries). PDPs have been instrumental in prioritising these types of products for the African Trypanosomiases, through the setting of minimal performance and operational characteristics that place product users at their core, aka Target Product Profiles.

This paper explores the models and contribution of three PDPs set up to deliver product innovation for the African Trypanosomiases (as part of larger disease portfolios): the Drugs for Neglected Disease Initiative (DNDi), the Foundation for Innovative New Diagnostics (FIND) and the Global Alliance for Livestock Veterinary Medicines (GALVmed). Drawing on empirical research and documentary data, we describe their working models, explore their product portfolios, and set out the stories behind some of their most high profile projects.

Our analysis reveals that PDPs are not static entities fulfilling singular product development functions. Rather, they are heterogeneous and evolving organisational forms. Having served up much of the ‘low hanging fruit’ from the product pipelines in their early years, they are being driven to transform by a number of factors, including donor pressures for greater impact and value for money, and the changing nature of R&D partnerships.

### The Emergence of Product Development Partnerships

While minimal collaboration between the public and private sectors had been the norm up until the mid-1970s, in the late 1970s and early 1980s, disillusionment with the state and the rise of a neoliberal agenda carved out a bigger space for the private sector. The latter half of the 1990s witnessed a burgeoning of Public-Private Partnerships (PPPs) for health. Conceived with the aim of overcoming market and policy failures, such collaborations leveraged new financial resources and proposed innovative solutions to intractable global health problems. A continued appetite for these “networked approaches” [16] (p. 2) into the first and second decades of the twenty-first century can be explained by three factors: the global burden of disease [17]; unprecedented levels of funding for health favouring the PPP approach (including from philanthropic and private sources) [18]; and new developments in the science and technology sectors [19].

Under the umbrella of PPPs for health, sub-categorisations have emerged, including: product-based or access PPPs, which seek to extend access to existing products for which there exists limited demand/ability to pay); issues or systems-based PPPs, which might, for instance, include PPPs seeking to improve health system capacity; and product development-based or PDPs [17]. The focus of this paper is largely on the third category, although the PDPs included in this review also provide some functions of access PPPs.

While Moran et al. sought to define PDPs using the criteria cited above, they also underlined that their summary definition—while expressing many of the shared characteristics of the PDP model—failed to convey the many differences that can still exist between different PDP entities [14]. For instance, while some have in-house laboratory and manufacturing capacity, others depend entirely on collaboration with others for these functions; and while some PDPs limit their operations to product development, others pursue additional objectives, including capacity building and technology transfer. Subsequently, identifying the key differences between PDPs is an important first step in understanding their scope and contribution.

Over the past ten years, a sea change has occurred in how PDPs for health are viewed. Once perceived as an unknown quantity [20], today, they occupy a central position in the global health infrastructure, and are credited as the main driving force behind an “unrecognised revolution in global health” [21]. To expand, Policy Cures noted that over a period of 15 years 485 neglected disease product candidates had entered the product pipeline, 58% of which came from PDPs and other PPPs [21] (p. 1). In addition, “Because these groups are driven by public health goals, their products are designed to be affordable and appropriate for developing countries” (ibid.). A review by Berdud, Towse and Kettler seconded this position [22]. Their work suggests that for malaria, at least, PDPs are one of the only incentivising mechanisms with a proven track record at this time.

In the new millennium, the case was made that a number of tropical infections, including HAT, should be taken forward as a group by virtue of their ‘neglected’ status and shared geographic overlap, and because cost savings and synergies could be levied if the different disease programmes acted together [23]. The resulting categorisation—Neglected Tropical Disease (NTD)—has subsequently become a successful “brand identity,” leveraging attention and resources for the named diseases [24,25].

The International AIDS Vaccine Initiative and the Medicines for Malaria Venture were the first PDPs to be established in the mid-1990s. In the new millennium, the Access to Essential Medicines and NTD movements helped catalyse the establishment of PDPs for NTDs. Prior to this, the World Health Organisation’s Special Programme for Research and Training in Tropical Diseases (WHO/TDR) had been “essentially the ‘only game in town’” for tropical disease for several decades [26] (p. 3).

In 2017, 14% (US$506m) of all R&D funding for neglected diseases was channeled through PDPs [27] (p. 99).

## 2. Materials and Methods

This paper reports on a mixed methods research project which employed a multiple case study design influenced by Yin and Blaikie [28,29]. We focused our research on the three PDPs that have an explicit focus on product development for the African Trypanosomiases. The objective of this study was to track the emergence and evolution of our PDPs over a prolonged period, noting the need to place empirical studies in a series of broader contexts if they are to be generalisable [30].

Between 2013 and 2019, a number of semi-structured interviews were undertaken with staff—past and present—from the three case study PDPs (*n* = 11); and with representatives from partner organisations, such as the Institute of Tropical Medicine, Antwerp; the Swiss Tropical and Public Health Institute; Dundee University’s Drug Discovery Unit; and the International Livestock Research Institute (*n* = 14). In addition, our research project joined the HAT Platform during the study period [31], providing opportunities to gain more informal insights into the work of DNDi and FIND.

Extensive notes were taken at conferences where the work of the foci PDPs was presented and discussed, for instance, DNDi’s tenth anniversary conference (Nairobi 2013), ISNTD D_3_ (London 2016), and the NTD Summit (Geneva 2017).

Finally, a discourse analysis of primary and secondary documentation was undertaken. This included strategy papers and annual reports produced by the PDPs themselves, and the academic and grey literature around PDPs more generally, for instance, the G-FINDER public search tool and reports, which collect funding data on R&D for neglected diseases [32]. We also drew on evaluation reports commissioned by some of the main donors currently supporting the PDP model.

Qualitative data (interview transcripts, primary and secondary documentation) were managed and analysed using NVivo—a software package for qualitative research. Quantitative data were managed in Microsoft Excel.

Ethics approval was attained from the University of Edinburgh’s formal ethics approval process in the School of Social and Political Science and the broader project was granted ethics approval by the European Research Council.

Work in relation to this output was conducted over an extended period. While our research project closed in February 2018, change in our case study organisations continued unabated. Therefore, in order to bring this paper up to date, we conducted follow up interviews where feasible (at GALVmed), and drew on other sources where not (e.g., conference notes, strategy papers and annual reporting). It is a limitation of our study that we were not able to conduct follow-up interviews at all three organisations.

## 3. Results: Product Development Partnerships for the African Trypanosomiases

### 3.1. Drugs for Neglected Disease Initiative (DNDi)

DNDi was launched in 2003 on the back of recommendations made by the Drugs for Neglected Diseases Working Group, a thinktank set up by MSF to investigate the crisis in R&D for neglected diseases. DNDi’s primary objective is to deliver new treatments for patients suffering from the most neglected diseases, through the development of new drugs or the reformulation of existing ones. Its original strategy was based on a “two-pronged approach to R&D,” which sought to address urgent need in the short term while attempting to deliver entirely new treatments in the long term [33] (p. 6). DNDi now operates a three-prong approach: managing short- (1–3 years), medium- (3–5 years) and long-term projects (6–15 years) [34].

DNDi is registered as a not-for-profit legal entity in Switzerland. It was founded by seven public and private institutions: MSF; the Oswaldo Cruz Foundation; the Indian Council for Medical Research; the Kenya Medical Research Institute; the Ministry of Health, Malaysia; Institut Pasteur, France; and WHO/TDR. It has headquarters in Geneva, and eight regional offices in New York, Kenya, India, Brazil, Malaysia, Japan, South Africa and the Democratic Republic of Congo.

DNDi’s early portfolio focused primarily on the kinetoplastid diseases, although its portfolio always remained open to other R&D projects where “glaring gaps” were apparent [33] (p. 3). In 2015, DNDi formally broadened its mission from neglected diseases to neglected patients, thus adopting “a more dynamic approach to the evolution” of its portfolio that has seen it take on paediatric HIV, Hepatitis C and antimicrobial resistance [34] (p. 10). DNDi’s 2019 portfolio tackles seven diseases and includes over 47 R&D projects. From this position, DNDi now aims to deliver 16 to 18 new treatments by 2023, and to ensure equitable access to these treatments. It has already delivered eight [34].

DNDi’s original not-for-profit R&D model was said to be characterised by four tenets: “a concretely patient-centred, needs-driven approach; a commitment to both equitable access to treatment for patients and open access to knowledge; financial and scientific independence; and the leveraging of existing knowledge and expertise by building solid alliances with public and private partners” [33] (p. 3). In 2019, its model is more clearly rearticulated as having six tenets: needs-driven; independent; collaborative, transparent and open; globally networked and access-orientated [34].

To expand on a few of these, DNDi has always prioritised a needs-driven approach to R&D by “beginning with the end in mind” [33] (p. 3). It has done this through the setting of Target Product Profiles (TPPs), in conjunction with other partners and, where possible, patient representatives. TPPs are a description of the ideal specifications needed for a given product, considering the needs of the patient and the main characteristics of the health system that serves them. Today, DNDi promotes the setting of “Public-Interest Target Product Profiles” in support of its needs-based agenda [34].

The main way DNDi maintains financial and scientific independence is through its funding policy, which seeks to prevent any single funder imposing undue influence or creating financial dependency. Accordingly, DNDi seeks to maintain a balance of public and private support, to minimise restricted funding, and to ensure that no one donor contributes more than 25% of the overall budget. DNDi has been much more successful at securing unrestricted funding than other PDPs, receiving 47% of its income as unrestricted contributions [34]. DNDi’s funding model does not require it to recoup R&D investments or finance future research through the sale of products or revenues generated by Intellectual Property. Instead, donor contributions pay upfront for R&D, delinking the financing of R&D from the price of the final product. In 2018, DNDi received Euros 66.4 million in multi-year funds (for DNDi and the Global Antibiotic Research and Development Partnership), as well as Euros 20.2 million in the form of in kind contributions from partners [35] (p. 3).

DNDi operates through a virtual R&D model, meaning it does not have any in-house laboratory or manufacturing capacity. This is why the building of successful alliances is so fundamental to its work. DNDi depicts its role as the “conductor of a virtual orchestra” [33] (p. 5), through which it draws on the capabilities and expertise of a host of partners straddling academia, public-sector research institutions, pharmaceutical and biotechnology companies, non-governmental organisations, other PDPs, and national governments. The PDP currently has 180 partnerships in over 40 countries [34] (p. 13). As of 2018, 34% of its partners were in low- and middle-income countries (LMICs) [34] (p. 21).

DNDi is committed to open and collaborative science, on which its operations depend. From 2012 to 2018, 57% of the compounds screened to evaluate their potential as drug candidates were made available to DNDi free of cost [35] (p. 7). It is through such arrangements that the PDP has begun to challenge the much-cited di Masi et al. (1991) figure, which puts the price of bringing a new drug to market at US$ 500 million. DNDi estimates it can develop and register a new treatment based on existing drugs for Euros 4–32 million and a novel chemical entity for Euros 60–190 million (attrition in the pipeline included) [34] (p. 18).

DNDi’s mission has always set out to utilise and strengthen R&D capacities in the countries where its portfolio diseases are endemic. Now, however, its ambition has grown, and it aspires “to contribute to new innovation eco-systems,” driven by scientific leaders in LMICs [34] (p. 22). This, it hopes, will change how research priorities are defined and where health R&D in the public interest is conducted. As part of this vision, the PDP has helped establish five disease-specific clinical research platforms. This includes the HAT Platform, established in the DRC in 2005, to build and strengthen treatment methodologies and clinical trial capacity. It is a common feature of the evolution of the PDPs under study that they seek to deepen and support LMIC leadership of disease control and elimination, primarily in recognition of the need to learn from and engage with the sites in which new technologies will be deployed.

In the past, drugs candidates for neglected diseases were progressed one at a time through a chain model. This was the model DNDi began with, but it quickly moved on (Simon Croft addressing ISNTD D3 conference 25 May 2016). Taking its cue from big pharma and other PDPs like the Medicines for Malaria Venture, DNDi switched to a portfolio model, whereby a pipeline is created with back-ups in place to replace, succeed or even synergise with other drug candidates already further along the development pathway. Table 1 depicts DNDi’s HAT portfolio.

#### Product Backstory: NECT, Fexinidazole and Acoziborole

DNDi’s three-pronged approach to R&D is well exemplified through its HAT portfolio. Initially targeting an urgent unmet need, the newly registered DNDi built on earlier work conducted by MSF Epicentre and WHO/TDR to move the HAT drug eflornithine into a combination therapy (to protect it from resistance and improve its efficacy). The chagas drug nifurtimox provided a new lead when, in 2006, it was registered for the compassionate treatment of melarsoprol-refractory HAT cases in combination with other trypanocidal drugs. Several trials were conducted, testing three different combinations. While all three combinations proved more efficacious than their monotherapy counterparts, the nifurtimox-eflornithine combination emerged as the safest option [36,37]. A multi-centre trial was initiated, with the results supporting the adoption of Nifurtimox-Eflornithine Combination Therapy (NECT) as the preferred treatment option for late-stage *T. b. gambiense* [38]. In 2009, NECT was added to the WHO’s Essential Medicines List, scoring an early success for DNDi.

Hoping to develop a wholly new treatment for HAT, DNDi carried out a major survey of previously discarded compounds known as nitroheterocycles for trypanocidal activity. A systematic review and profiling of more than 700 nitroheterocyclic compounds was undertaken in 2005. From this search, the nitroimidazeole compound fexinidazole was identified as a promising drug candidate [39]. In this manner, fexinidazole is said to have been ‘rediscovered’ by DNDi, as the drug (once known as Hoe 239) had previously been in preclinical development as a broad spectrum anti-protozoal drug by Hoechst AG, Frankfurt, Germany (now Sanofi, Paris, France) during the 1970s and 1980s. DNDi went on to develop fexinidazole in conjunction with Sanofi. The drug entered clinical development in September 2009. In November 2018, fexinidazole was approved by the European Medicines Agency as the first all-oral treatment for both stages of T. b. gambiense. In 2019, it was added to the WHO’s list of Essential Medicines.

Fexinidazole eliminates the need for systemic hospitalisation for late-stage patients and will reduce the number of lumbar punctures required for diagnosis and follow up. In short, fexinidazole proposes to change the paradigm of HAT care by making treatment simpler and safer. Nevertheless, within DNDi and the broader HAT community, there remains further momentum to improve HAT treatment. To explain, while a *good enough* drug might suffice for disease control, given the goal of reaching and sustaining HAT elimination, something more closely resembling a *perfect* drug is desirable. Fexinidazole is not considered a perfect drug, given concerns around dosage (once a day for ten days), and the fact that it has to be metabolised to work (Interview 30 March 2013). This again, is where DNDi’s portfolio model comes into play, driving the PDP to explore several back-up compounds, even as fexinidazole progressed through clinical trials.

Between 2007 and 2008, DNDi worked to establish the HAT Lead Optimization Consortium (in conjunction with SYNEXIS, and Pace University), which seeks to optimise new classes of compounds identified from screening activities. In 2008, DNDi was approached by the biotech company Anacor Pharmaceuticals Inc., Palo Alto, CA, United States, with a promising new chemical series known as oxaboroles. In 2009, it opted to advance the compound SCYX-7158—now known as acoziborole—into preclinical development [33].

Acoziborole is an entirely novel chemical entity with the potential of becoming the first oral, single-dose treatment for *T. b. gambiense*. The drug is currently in a Phase II/III study in the Democratic Republic of Congo, with results expected by the end of 2020 [35].

### 3.2. Foundation for Innovative New Diagnostics (FIND)

FIND was launched at the World Health Assembly in May 2003. Its purpose is to develop and deliver innovative, field-adapted diagnostics for use in low-resource settings. FIND is an independent Swiss Foundation, established as a not-for-profit legal entity. It has its headquarters in Geneva and four regional hubs in India, Kenya, South Africa and Vietnam.

Initially focused on tuberculosis, HAT was the first NTD to be included in FIND’s portfolio (in 2006). FIND’s ‘NTD portfolio’ was subsequently expanded to include leishmaniasis (in 2010) and chagas disease (in 2012). It has since been augmented further to include buruli ulcer and schistosomiasis. Inclusive of NTDs, FIND’s portfolio incorporates four main programme areas (tuberculosis and acute febrile respiratory infections; malaria and acute febrile syndrome; Hepatitis C) and several mini portfolios. Under its current strategy (2015–2020), FIND is attempting to catalyse the development of 15 new diagnostic technologies [40]; ten of which have already completed all phases of development [41] (p. 9).

In 2010, FIND underwent something of a crisis, having accumulated a significant financial deficit that served to raise serious concerns around the organisation’s governance and management structure (“Original accounting policies had led to an inappropriate recognition of income and the reinstatement of income revealed an accumulated deficit of USD 6.03 mill in 2010, which was mostly due to overhead spending without related funding base” [42] (p23)). The situation was so grave that questions were raised around whether FIND should continue to operate at all, or whether it should be merged with another PDP. This did not happen. Instead, between 2011 and 2013, FIND restructured (becoming a “lean, expert organization” that outsources work to consultants) [40] (p. 35), overhauled its financial systems, and rearticulated its role in product development. Today, FIND is again deemed “a viable PDP” [42] (p. 18).

**Figure 1:**
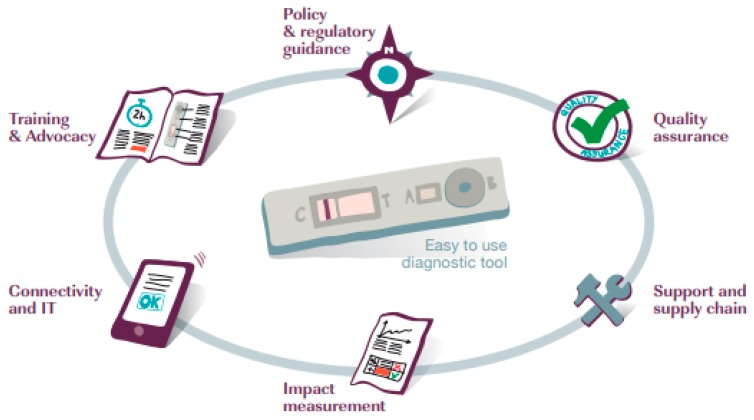
Elements of a complete diagnostic solution [40] (p. 23).

In its new incarnation, FIND is guided by a five-year strategy that has placed greater emphasis on its roles as ‘mobilizer’, ‘bridge builder’, and ‘enabler’ [40]. It has moved away from a primary focus on developing individual diagnostic technologies in conjunction with private companies to supporting complete diagnostic solutions, termed *packages*. This revision recognises that a diagnostic test requires a suite of ingredients to support its roll out and use, in what are often weak health systems (see Figure 1).

Given this premise, FIND now works around four strategic goals: (1) **catalyse development** by identifying needed diagnostic solutions and removing barriers to their development; (2) **guide policy and use** by leading products through the clinical trials pathway to global policy on use and market entry; (3) **accelerate access** by supporting uptake and appropriate use of diagnostics to achieve health impact (4) **shape agenda** by improving understanding of the value of diagnostics and strengthen the commitment to their funding and use [40].

Like DNDi, FIND operates through a virtual model, relying on partners to realise its goals. Where once the PDP operated through bilateral partnerships, under its current strategy it aspires to adopt coalition- and initiative-based approaches, in order “to achieve broader reach and stronger outcomes” [40] (p. 36). FIND also hopes to establish more output-orientated partnerships, for example, with other PDPs “where alignment of disease-level strategies is pursued and development of complementary, rather than duplicative or conflicting initiatives is encouraged” (ibid). It is thought that such collaborations would facilitate joint funding and implementation opportunities.

FIND’s donors include the Global Fund to Fight AIDS, tuberculosis and malaria, the UK’s Department for International Development (DFID), the Bill and Melinda Gates Foundation (BMGF), and the Australian Department of Foreign Affairs and Trade. In 2017, FIND’s funding from donors was nearly US $56 million, with a further US $3.9 million given as in-kind donations [41] (annex p. 8). Unlike DNDi, FIND does not have an explicit policy to prevent one donor emerging as dominant, and in its early years found itself heavily dependent on funding from the BMGF. That said, it was able to achieve significant funding diversification in the period of 2009–2013, and is now in a less precarious position [42]. FIND has had some success in reducing the proportion of restricted funding it receives (bringing the total down from 100% in 2010) but it has not been as successful as DNDi [41,42].

FIND’s portfolio (see Table 2) for HAT is based on two objectives: (i) increase detection of HAT through improved case finding; and (ii) facilitate faster, less burdensome confirmation of HAT through improved tools [40].

#### Product Backstory: Competing Rapid Diagnostic Tests for HAT

The Institute of Tropical Medicine, Antwerp (ITM) enjoyed a de facto monopoly over HAT diagnostics for several decades years prior to the establishment of FIND in 2003. The CATT was developed at ITM, and launched in 1978. Yet, it was not adopted at scale until the mid-1990s. Our fieldwork revealed that the right people had to be persuaded of the scientific merit of the CATT in order to abandon tried and tested techniques. That this took nearly 20 years points to missing elements in the innovation puzzle for HAT prior to the establishment of PDPs: product promotion and access (Interview 24 June 2015). FIND, in its current incarnation, recognises this fact through its endorsement of complete diagnostic solutions.

In the new millennium, FIND and ITM initially set out to work together to develop a rapid diagnostic test (RDT) for HAT, but when disagreements arose between the two parties, ITM determined to develop a competing test in parallel (Interview 19 November 2013). This period of competition proved fruitful, and in 2012 and 2013, two new RDTs for HAT were launched: the SD BIOLINE HAT, co-developed by FIND and manufactured by Standard Diagnostics, Suwon, S. Korea; and ITM’s HAT Sero K-SeT, manufactured by the Coris BioConcept, Gembloux, Belgium.

In theory, ITM boasted certain advantages that should have seen its test dominate: ITM manufactures the native antigens used in both tests (meaning in theory it can set a ceiling on how many tests FIND can produce); it is also the WHO collaborating centre for HAT diagnostics. However, FIND has proved an astute political player. At its product launch in December 2012, FIND formally credited ITM with helping develop the test, glossing over their rivalry to paint the relationship as collaborative (Interview 19th November 2013). It has also carved out a role for its RDT in low areas of low endemicity (knowing the ITM test would be used in areas of high endemicity), framing its usefulness in support of elimination efforts [43]. Finally, FIND secured a lower unit price for its tests (less than US $1 per test as opposed to ~ US $2.50) (Interview 19 November 2013) [44], thus contributing to a narrative—that its research arm helps to perpetuate—that the SD RDT is notably cost-effective [45]. Implementation research conducted by the Diagnostic Tools for HAT Elimination and Clinical Trials project (DiTECT-HAT) has since suggested that the ITM and FIND RDTs do not even necessarily need to compete; that potentially, they can both be incorporated into algorithms for passive case detection and post-elimination monitoring [46]. This is because while both tests use the same native antigens, their separate development pathways have produced RDTs with slightly different performance characteristics in field settings that enable them to identify overlapping but nevertheless immunologically different patient populations.

In recent years, both FIND and ITM have continued to innovate, setting out—again, in parallel—to develop second generation RDTs for HAT based on recombinant antigens. And while our research suggests that the relationship between ITM and FIND has at times been privately strained, the external view of the ensuing rivalry has been largely positive, suggesting competition is both a fertile catalyst of innovation and a means of securing production:

“A healthy competition exists between several groups to develop HAT rapid diagnostic tests, which should improve quality and provide options in case one test fails to improve diagnostic accuracy. As a neglected tropical disease and with decreasing disease prevalence, progressive commercial disinterest is a possibility for HAT. Thus, reliance on a single rapid diagnostic test for which production could be stopped for economic reasons is dangerous, and could harm the elimination strategy”.

[47] (e306–e307)

Indeed, the heightened activity in this field has since attracted other players to explore HAT diagnostics [48]. In short, the establishment of FIND has done much to energise a field that had for several decades, lain dormant.

### 3.3. Global Alliance for Livestock Veterinary Medicines (GALVmed)

GALVmed, formerly the Global Alliance for Livestock Vaccines (GALV), was launched in 2004. GALVmed is set up to make livestock medicines, vaccines and diagnostics accessible and affordable to farmers in developing countries. GALVmed’s goal is to develop and advocate for the benefits of animal health products to meet the needs of small-scale livestock producers, passing them onto others to manufacture and distribute. Trypanosomiasis has been an important strand of GALVmed’s work since the beginning. Besides the impacts of livestock health, mortality and productivity, an important element of this work would be to reduce the role of livestock as a reservoir of the zoonotic *T. b. rhodesiense* form of sleeping sickness [49].

Since its inception, the majority of GALVmed’s funding and all of its core funding has come from DFID and the BMGF. GALVmed (then GALV) had been established to explicitly focus on vaccines but this rapidly expanded to include other veterinary products. Between 2005 and 2017 DFID and the BMGF provided almost US $60 million of support (DFID project record [50]). Initially DFID has been the majority funder but this balance of funding responsibility has gradually reversed. In 2011, DFID awarded an additional grant of just over US $10 million to support the development of an integrated package of tools and policies for the cost-effective control of animal African trypanosomosis (AAT). This package aspires to deliver a new field diagnostic tool and new trypanocidal drugs, and to establish the basis for the development of a vaccine for AAT (DFID project record). The BMGF has since complemented this grant twice in support of project Phase 1 and Phase 2 (the BMGF awarded US $1.4 million in January 2014). The stated purpose of the BMGF grant was to develop safe and effective drugs against drug-resistant AAT, to develop a diagnostic test for field diagnosis in cattle, and to improve quality control of existing trypanocidal drugs used by smallholder livestock keepers in sub-Saharan Africa [51].

GALVmed’s early work included the development of an anti-trypanosomiasis vaccine candidate, but this failed to progress beyond exploratory stages due to numerous technical difficulties (Interview 27 March 2013). Over the past ten years, GALVmed’s work has evolved. Generally, there has been a significant shift away from R&D, through testing of pilot field projects to assess commercial development, towards the current establishment of large-scale, market-based initiatives. In a similar vein to the other PDPs, GALVmed now sees its work as having two strands. One remains new product development, with perhaps a greater focus on improving products to meet the specific needs of smallholder farmers. The second is commercial development—utilising the commercial value of products to ensure their sustainable availability to smallholder farmers.

The ‘product development’ strand has also broadened (See Table 3 [52]). Product improvement requires working with different stakeholders, including more directly with smallholders. Product development is increasingly delivered through complex consortia, as can be seen with GALVmed’s AAT programme.

In response to technical difficulties in the creation of an effective vaccine against AAT, GALVmed diversified its AAT portfolio. Its multi-pronged approach, which placed an emphasis on improving the systems through which animal health technologies would be delivered, required a different approach to partnerships. GALVmed now facilitates more than 20 often-complex research partnerships that include global pharmaceutical companies, universities, public research groups and organisations representing farmers. This has led to the current state of affairs with the marketing of a pen-side diagnostic test by a commercial animal health company and the trypanocide moving into development with a commercial partner.

Over the last decade we can see a tangible shift in GALVmed’s approach [53]. It is now less concerned with picking ‘low hanging’ technological fruits—many of which have been picked—and more concerned with understanding the needs of smallholders, delivering sustainable services and advocating for better policy to ensure appropriate products are developed and delivered. This means understanding the complex interplay between retailers, veterinary services and smallholders in multiple contexts, engaging much more in policy circles, and giving more thought to market needs and commercial concerns.

This broader approach to the delivery of animal health products is mirrored to an extent by GALVmed’s two main donors, the BMGF and DFID. The BMGF and DFID recently (2018) funded a new US $50 million programme with GALVmed called VITAL (Veterinary Innovations Transforming Animal Health and Livelihoods) which aims for sectoral transformational change—focusing on the development of six new vaccines and partnering with the animal health industry to develop five large-scale product development networks in Asia and Africa.

In one key respect, animal health product delivery is markedly different from human health: there is more potential for commercial profit in delivering new products. This is often much less clear, especially for NTDs like HAT, given their close relationship to poverty. GALVmed is therefore much more interested in understanding the commercial context in which new health products will exist than either FIND or DNDi. Indeed, GALVmed sees itself as having a catalytic role to “educate industry that LMICs are interesting” (Interview 8 August 2019). GALVmed’s trypanosomiasis programme in particular is focused on consortium building given its immediate focus on a pen-side test and new drugs. GALVmed’s consortium building and commercial engagement, ostensibly to ensure the immediate commercial viability of its products, can play an important role in future-proofing its activities. Many of the most pressing future concerns in the livestock sector in developing countries—population growth/demand for high quality protein due to an emerging middle class, the rise of antimicrobial resistance, and the impacts of climate change—have strong commercial drivers that GALVmed could be well-placed to engage with to lever the delivery of new technologies.

#### Product Backstory: Pen-Side Diagnostic Test

Drugs to treat AAT do exist, but most were introduced over 40 years ago. One strand of GALVmed activity is focused on incentivising pharmaceutical companies to search for new classes of trypanocides [3]. Another important strand of work is the development of more effective diagnostic approaches. There are no specific clinical signs of infection by *T. vivax*, *T. congolense* or *T. b. rhodesiense*, so differential diagnosis is difficult. Currently, the only way to confirm a diagnosis in infected animals is to demonstrate and identify the parasites in blood and other bodily fluids (which is in itself difficult as parasite numbers are generally low). Most treatment of AAT is therefore presumptive, which has costs for farmers and their perception of the efficacy of treatment [54,55]. The lack of a simple diagnostic test similarly makes it difficult to identity and deal with parasitic reservoirs within livestock populations. Developing a simple pen-side diagnostic test, focused on *T. b. rhodensiense*, may therefore have implications for both animal and human health [56].

GALVmed funded a number of laboratories with different approaches to antigen discovery in a competitive manner. This portfolio approach preserved the diversity of promising antigen candidates that were discovered and could then be tested. One of the partners included the University of Dundee where promising *T. vivax* proteins were identified and developed into prototype lateral flow and ELISA tests [54]. A further collaboration with BBI Solutions, who specialise in immunoassay development, led to the development of a simple lateral flow test that could be used in situ as the favoured approach (hence ‘pen-side’). This required no electricity, additional equipment or diagnostic expertise. Not coincidentally, the BBI Solutions-Dundee partnership was built on earlier work in relation to the development of a lateral flow diagnostic test for HAT, supported by FIND. GALVmed also worked with the pharmaceutical company CEVA, the University of Bordeaux, the French research agency CIRAD and teams of researchers from across Africa to develop the first commercially available rapid field diagnostic test against AAT, a lateral flow test, which can detect infection with both the *T. congolense* and *T. vivax* strains. This is now being used in several regions in Cameroon.

These new generation tests are not perfect. In particular, they remain expensive (currently more expensive than simply treating a cow so there are issues as to whom they are of utility to). The cost of the test means that it may currently be of more interest to academics. And the targeting of the tests on *T. congolense* and *T. vivax* means they are not a tool of utility to reduce *T. b. rhodesiense* parasite burdens within cattle as a means of reducing the risk of human infection. GALVmed has, however, demonstrated its ability to build a new diagnostic pipeline from lab to livestock.

## 4. Discussion: Innovation, Evolution and Sustainability

To date the majority of studies on PDPs have examined them only as relatively static entities [14,57], noting their differences from one another with less emphasis on their progression over time. In this article, we attempted to sketch the emergence, evolution and contributions of three PDPs closely associated with the African trypanosomiases. All three have delivered for the archetypal neglected tropical disease and been changed in the process.

DNDi changed tack early on to mirror the portfolio approach of big pharma, building a product pipeline for HAT for the first time in history. At the same time, it broke with the profit-driven model of industry—delinking R&D from the price of products—to assert that product development in the public interest is possible. FIND’s entry into HAT diagnostics shook up a field that had lain dormant for decades. Its early work contributed to the development of competing, yet complementary screening tests that will help secure the supply chain for elimination, and hopefully drive further advancements that could help simplify HAT’s complex diagnostic tree [58]. GALVmed cast off its (inherited) vaccine pursuit to prioritise a more user-focused product portfolio. In doing so, it produced a successful pen-side test even if it was not able to stick to the ‘cost’ component of its target product profile. This achievement has provided a proof of concept for its approach, which should help drive further projects, and hopefully donor confidence.

All three PDPs have also evolved in the sense that they have expanded their portfolios and extended their missions, moving away from a narrow focus on product development to encompass a broader suite of complementary functions to ensure products are accessible and impactful. Our research suggests all three case study PDPs are being driven to evolve by three interlinked factors: funding, an impetus to show impact and the changing nature of R&D partnerships.

The most important factor is funding—primarily to ensure sufficient investment in research and that incentives exist to encourage private sector actors to engage in product development. The years 2000–2010 are now viewed as a ‘golden era’ for global health funding. During that decade, development assistance for health grew at an annual rate of 11.2%. By contrast, from 2010–2017, the total growth was just 1% annually [59]. And where once PDPs were channeling around a quarter of all R&D funding for neglected diseases, in 2017, that proportion had dropped to 14% [27] (p. 99). While there are many external reasons to explain the constrained funding environment (the aftermath of the financial crisis, rising nationalism), it must be frustrating for PDPs, given that in many ways, they represent a ‘best buy’ in global health. From a donor perspective, not only are our case study PDPs productive, they reduce financial risk, incur lower R&D costs (through portfolio approaches), and present donors with lower management responsibilities than if they were to give to multiple product developers. Moreover, from the perspective of public sector investment, PDPs provide an avenue for public-funded basic research to feed into public goods and much-needed products.

However, where PDPs are less successful is in demonstrating impact, at least in the short term. During their formative years, all three organisations were incentivised by their major donors to deliver new technological innovations. There were set product delivery targets and focused on delivering new innovations above all else. This resulted in multiple new products that would not have been taken forward by industry. However, a move towards what we term a ‘post-low hanging fruit phase’ characterises DNDi, FIND and GALVmed. In short, the easy wins have been achieved. Increasingly, PDPs are being asked to show not just outputs but outcomes as donors have become more concerned with measuring the impacts of their investments. Yet, the type of health products that our case study PDPs produce—drugs, diagnostics and vaccines—tend to incur long lead times to impact (there is a long development times for drugs and vaccines, and uncertain patient pathways from diagnostics to treatment). Moreover success in these fields is an anomaly rather than a given, hence why DNDi has to factor ‘attrition’ into its drug development calculations. Donors clearly have a concern with value for money and impact, as is demonstrated by the growing number of donor evaluations into the PDPs they support [42,60].

Each of our case study PDPs exists in an ever more complex institutional context. Traditional institutions jostle for funding and prioritisation—and the NTD sector is a good example of this [25]. Meanwhile, new actors emerge as bilateral funders like DFID increasingly look to experiment with private sector approaches and work through other organisations, given concerns around the ability of traditional aid to drive efficiency, value and scalability [61]. Funders like the BMGF are now providing more funding directly to manufacturers [40,42]. FIND has flagged this up, pointing to the “increased but fragmented” participation in product development [40] (p. 19). There are potentially advantages to this—more opportunities for collaboration, information sharing and building a portfolio-driven approach, but also disadvantages—increased transaction and opportunity costs, imperfect access to information hampering decision-making, and collaboration turning into competition if budgets are tight. There needs to be an important role for donors in this complex, evolving environment. Giving incentives and impetus to working across PDPs can create new opportunities—witness the GALVmed lateral flow diagnostic test genesis described above—but the “absence of an overarching and sustainable framework to govern and drive public interest R&D” threatens to hamper progress [34] (p. 38).

PDPs find themselves in a bind. The three PDPs described here have all developed innovations (although it is too early to assess their impacts). It can be argued that each has already picked its ‘low-hanging fruit’ and future innovations will be more expensive and harder work to deliver. They all operate in a funding environment which is contingent on a very small number of donors, who are increasingly focused on results, impacts and managing risk [42]. There is a tension here between ability to deliver and expectation of impact, and the response of the PDPs has been to diversify the breadth of their disease portfolios and the nature of their partnerships. Allied to this, donors are exploring other private sector delivery mechanisms, forcing PDPs to re-position themselves as ‘brokers’, ‘enablers’ and ‘interpreters’ within this new, ever more complex global health innovation arena; the end goal being to diversify their funding base by demonstrating their value to the private sector and venture capitalists.

## 5. Conclusions

It is too soon to judge the contributions of DNDi, FIND and GALVmed to the elimination of African Trypanosomiasis. Most of the products described here are too new or still under trial and in any case, it would be an enormously complex question to answer given technological innovations can only ever be a contingent component of any successful health intervention or system. Instead, this paper has attempted to describe the broader role of PDPs as deliverers of new tools to combat the archetypal “tool-deficient” NTD. New tools have and are being delivered. It is unclear that these new tools would have been delivered so quickly without the role of PDPs to broker, connect and fund research and innovation.

PDPs have also helped (in conjunction with WHO and other partners) rebuild and reinvigorate the epistemic community around tropical medicine. This is perhaps the biggest shake up of that network since it first emerged during the colonial period [4]. PDPs have helped attract big pharma back into this network, but even more importantly, they have helped formalise the central role played by public bodies in disease endemic countries. The HAT platform established with the help of DNDi, is a case in point [31].

PDPs have already contributed to the global campaign to see HAT eliminated as a public health problem by 2020—in delivering new diagnostics and treatments—but their larger contribution is yet to be realised: delivering at scale the products that could see the transmission of HAT eliminated by 2030. Reaching and sustaining this goal requires HAT services be integrated into national healthcare structures. This paradigm shift is complex to engineer—but from a technical perspective, it is only made possible by the development of new, safe, easy to use products that can be deployed at point of care. PDPs have been instrumental in prioritising these types of products for the trypanosomiases, through the setting of performance and operational attributes that place patients and product users at their core. It is telling that PDPs like DNDi, FIND and GALVmed are broadening their partnership networks and integrating their activities into nascent health and pastoral systems. This can facilitate innovations more closely tailored to the needs of countries, clinics and farms. It can also facilitate spillovers between PDPs and other actors. There is a danger, however, that PDPs—in trying to do too much to stay relevant to donors—could lose their important singular focus. This is a lesson for donor, PDP and policymakers alike if the elimination of African trypanosomiasis is to not only be attained, but sustained.

## Figures and Tables

**Table 1:** DNDi’s HAT portfolio.

Research >	Translation >	Development >	Implementation
Oxaborole SCYX-1330682		Acoziborole	Fexinidazole for T. b. gambiense
Oxaborole SCYX-1608210		Fexinidazole for *T. b. rhodesiense*	Nifurtimox-Eflornithine Combination Therapy (NECT)

**Table 2:** FIND’s HAT portfolio.

Catalyse Development >	Guide Use and Policy >	Delivered
HAT/Malaria combo RDT	2nd generation rapid diagnostic test (RDT), using recombinant antigens	1st generation RDT, using native antigens
		Primo Star iLED fluorescence microscope (iLED FM)
		Loop-mediated isothermal amplification (LAMP) of DNA

**Table 3:** GALVmed’s programme objectives for Animal African Trypanosomaisis.

Objective	Activities
The development of a new class of trypanocides for therapeutic and prophylactic field use.	Drug discovery work is taking place through a substantial network of partners and is the major focus of the Tryps 2 programme. The specific objectives of this work are one therapeutic candidate and one late-stage prophylactic compound to be transferred to a commercial company for full development. Additionally, a comprehensive backup pipeline of compounds to be made available.
The development of a pen-side diagnostic for field use.	One new pen-side diagnostic test will be licensed to a commercial company for subsequent production and marketing.
The development of improved integrated control methods at the farm/village level resulting in more effective use of the full range of vector and disease control measures.	Under the Tryps 2 programme, continuing support for improved trypanocide regulation and quality control is being provided through capacity building activities in two African laboratories.
Improved quality and regulatory control of trypanocides in Africa to counter the growing problem of counterfeit and substandard drugs.	Developing a better understanding of the integrated use of diagnostics, trypanocides, trypanotolerant breeds and vector control methods at the farm/community level in different farming and eco-systems.

## Abbreviations

AAT	Animal African Trypanosomiasis
BMGF	Bill and Melinda Gates Foundation
CATT	Card Agglutination Test for Trypanosomiasis
CIRAD	French Agricultural Research Centre for International Development
DFID	UK’s Department for International Development
DNDi	Drugs for Neglected Diseases Initiative
FIND	Foundation for Innovative New Diagnostics
GALV	Global Alliance for Livestock Vaccines
GALVmed	Global Alliance for Livestock Veterinary Medicines
HAT	Human African Trypanosomiasis
ITM	Institute of Tropical Medicine, Antwerp
LMICs	Low- and Middle-Income Countries
MSF	Médecins Sans Frontières
NECT	Nifurtimox-Eflornithine Combination Therapy
NTD	Neglected Tropical Disease
PDP	Product Development Partnership
PPP	Public-Private Partnership
R&D	Research and Development
RDT	Rapid Diagnostic Test
SD	Standard Diagnostics
TPP	Target Product Profile
WHO/TDR	World Health Organisation’s Special Programme for Research and Training in Tropical Diseases
